# Characteristics and Unexpected COVID-19 Diagnoses in Resuscitation Room Patients during the COVID-19 Outbreak—A Retrospective Case Series

**DOI:** 10.1155/2020/2721381

**Published:** 2020-08-17

**Authors:** Sebastian Bergrath, Olaf Aretz, Hendrik Haake, Adrian Ringelstein, Ingo Greiffendorf, Ullrich Graeven, Jochen Windfuhr

**Affiliations:** ^1^Zentrum für klinische Akut-und Notfallmedizin, Kliniken Maria Hilf, Akademisches Lehrkrankenhaus der RWTH Aachen, Mönchengladbach, Germany; ^2^Lehrstuhl für Anästhesiologie, Medizinische Fakultät der RWTH, Aachen, Germany; ^3^Klinik für Kardiologie und Internistische Intensivmedizin, Kliniken Maria Hilf, Akademisches Lehrkrankenhaus der RWTH Aachen, Mönchengladbach, Germany; ^4^Klinik für Radiologie und Neuroradiologie, Kliniken Maria Hilf, Akademisches Lehrkrankenhaus der RWTH Aachen, Mönchengladbach, Germany; ^5^Klinik für Hämatologie, Onkologie, Gastroenterologie und Infektiologie, Kliniken Maria Hilf, Akademisches Lehrkrankenhaus der RWTH Aachen, Mönchengladbach, Germany; ^6^Klinik für Hals-Nasen-Ohrenheilkunde, Kliniken Maria Hilf, Akademisches Lehrkrankenhaus der RWTH Aachen, Mönchengladbach, Germany

## Abstract

**Introduction:**

Emergency department (ED) triage regarding infection with severe acute respiratory syndrome coronavirus-2 (SARS-CoV-2) is challenging. During the coronavirus disease 2019 (COVID-19) outbreak in Germany, the diagnostic outcomes of critically ill patients admitted to the resuscitation room in the ED of our academic 754-bed hospital should be analyzed.

**Methods:**

All resuscitation room patients between March 1st and April 15th 2020 were included in this retrospective study. Every patient with suspicion of SARS-CoV-2 infection received a pharyngeal swab for real-time polymerase chain reaction (rt-PCR), divided in the clinical subgroups of “highly suspicious for COVID-19” and “COVID-19 as differential diagnosis.” All respiratory and infectious symptoms were included as at least “differential diagnosis” as an expanded suspicion strategy.

**Results:**

Ninety-five patients were included (trauma *n* = 14, critically ill *n* = 81). Of 3 highly suspicious patients, 2 had rt-PCR positive pharyngeal swabs. In 39 patients, COVID-19 was defined as differential diagnosis, and 3 were positive for SARS-CoV-2. Of them, pharyngeal swabs were positive in 1 case, while in 2 cases, only tracheal fluid was rt-PCR positive while the pharyngeal swabs were negative. In one of these 2 cases, chest computed tomography (CT) was also negative for ground-glass opacities but showed a pulmonary abscess and pulmonary embolism.

**Conclusion:**

We recommend an expanded suspicion strategy for COVID-19 due to unexpected diagnostic outcomes. Personal protective equipment should be used in every resuscitation room operation due to unexpected cases and initial knowledge gaps. Furthermore, tracheal fluid should be tested for SARS-CoV-2 in every intubated patient due to cases with negative pharyngeal swabs and negative chest CT.

## 1. Introduction

In February 2020, the coronavirus disease 2019 (COVID-19) reached the German region of Heinsberg. Our 754-bed academic teaching hospital is located in the nearby urban region, serving as the regional trauma center, supraregional comprehensive stroke center, cardiac arrest center with capability for extracorporal cardiac life support (ECLS), and ear-nose-throat clinic. Triage regarding COVID-19 in the emergency department (ED) is difficult due to changing recommendations by the German Robert Koch Institute and nearly daily new published knowledge and clinical experiences during this time period [1–4]. Especially, resuscitation room management of critically ill and trauma patients throughout the COVID-19 pandemic is challenging due to new safety measures and procedures as well as patients that are often not able to communicate or are already intubated by the emergency medical service. Additionally, medical treatment of critically ill patients with a previously unknown disease and missing experiences in management of such patients may lead to insecurities and necessary adaptions of processes.

The aim of this study was to analyze the characteristics and clinical features of resuscitation room patients during the COVID-19 outbreak in our region, especially those with suspicion of COVID-19. The rate of confirmed infections with severe acute respiratory syndrome coronavirus-2 (SARS-CoV-2) and the clinical characteristics at ED admission of this collective should be evaluated. Diagnostic outcomes regarding infection with SARS-CoV-2 including unexpected cases of COVID-19 should be extracted and analyzed in detail to allow future risk stratification in the resuscitation room. To our knowledge, no prior studies evaluated the collective of resuscitation room patients during the COVID-19 pandemic in Germany.

## 2. Methods

### 2.1. Study Design and Setting

We conducted a retrospective case series of all resuscitation room patients between March 1st and April 15th 2020 in our urban ED with 40000 annual visits (Kliniken Maria Hilf, Mönchengladbach, Germany).

### 2.2. Data Sources

Data of resuscitation room patients were extracted for quality management purposes routinely since August 2018. After each resuscitation room patient, a standardized questionnaire about conducted procedures was filled out, and data was transferred into a secured database, enabling periodically quality circles and reports. This database was used to identify the cases to be included. Admission diagnoses, conducted procedures, and criteria for COVID-19 suspicion were extracted from the electronic health records (IMedOne, Deutsche Telekom Healthcare and Security Solutions, Bonn, Germany). Diagnostic outcomes of conducted real-time polymerase chain reaction (rt-PCR) were captured from the laboratory reports within the clinical information system (IMedOne, Deutsche Telekom Healthcare and Security Solutions, Bonn, Germany). This way also laboratory and radiologic results were extracted.

### 2.3. Suspicion Criteria and Diagnostic Workup

All patients with clinical suspicion of COVID-19 received a pharyngeal swab in the resuscitation room and rt-PCR for SARS-CoV-2 in an external laboratory (Labor Stein, Mönchengladbach, Germany). Suspicion of COVID-19 was defined as respiratory symptoms and/or fever and malaise in the beginning of the study period, and adherence to Robert Koch Institute guidelines (Berlin, Germany) was mandatory [5]. Due to increasing numbers of COVID-19 in Germany and modified recommendations by the Robert Koch Institute over time, every patient with respiratory symptoms/compromised respiratory parameters and every signs of general infections were defined as suspicious for COVID-19 in the second half of the study period. Therefore, we used broader suspect case criteria, and all patients that had any form of impaired respiratory function were defined as potentially suspicious for COVID-19. Consecutively, these patients were handled with personal protective equipment, and every endotracheal intubation was performed with 3 filtering face masks for suspected cases and 2 filtering face masks for other patients that needed to be intubated in the ED.

### 2.4. Outcomes

**Figure 1:**
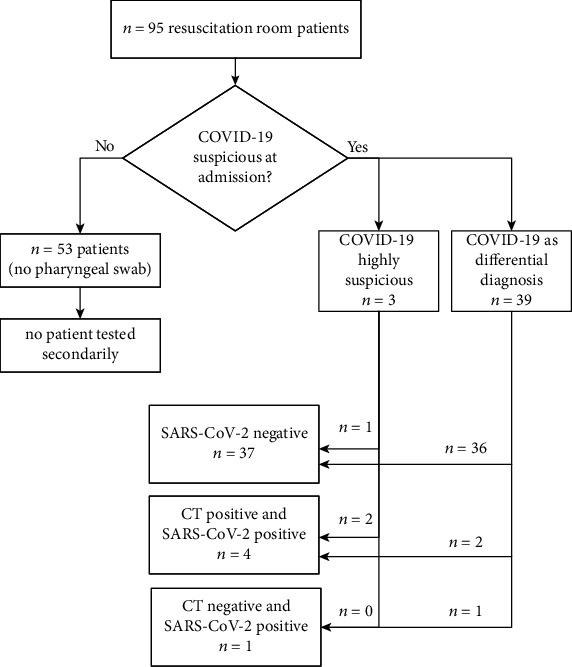
Study flow chart and main results. COVID-19: coronavirus disease 2019; SARS-CoV-2: severe acute respiratory syndrome virus 2; CT: chest computed tomography.

Patient characteristics of resuscitation room patients at the time point of ED admission and the subgroup of suspected COVID-19 cases were analyzed retrospectively. We evaluated demographics, vital signs, body temperature, and blood gas analysis parameter including serum lactate for all resuscitation room patients at the time point of ED admission. For SARS-CoV2 positive patients, we analyzed also white blood count, C-reactive protein, d-dimers, lymphocyte count, and procalcitonin at this time point. Suspected COVID-19 cases were divided in two groups, highly suspicious cases and COVID-19 as differential diagnosis (Figure 1). In unexpected confirmed SARS-CoV-2 infections, critical case analyses were performed. Critical care measures conducted in the ED were analyzed in respect to clinical signs of COVID-19 and computed tomography (CT) findings. CO-RADS classifications 1–6 were used to graduate to radiographic findings of chest CT [6, 7]. CO-RADS 1 and 2 were defined as negative, 3 to 5 as positive regarding COVID-19.

### 2.5. Ethics, Data Privacy, and Study Registration

All analyses were conducted with already-collected routine data. No additional data was acquired. Approval by the ethics committee of the university hospital Aachen, Germany (ethics committee registration number EK 146/20), was obtained prior to data collection and analysis. The study was registered in the Clinical Trial Center Aachen, Germany (registration number CTC-A 20-165).

### 2.6. Statistics

Descriptive statistics were conducted with Prism 8 (GraphPad Software, San Diego, USA).

## 3. Results

Overall, 95 patients (43% female; mean 2.1/day; range 0-5/day) were treated as resuscitation room patients between March 1st and April 15th 2020. Major trauma was the reason for admission in 14 patients (14.7%), and nontrauma resuscitation was performed in 81 (85.3%) patients. Clinical characteristics at the time point of admission of all patients are summarized in Table 1.

In 3 patients, a strong suspicion of COVID-19 was assumed at admission (Robert Koch Institute “suspected case”), 2 of them were diagnosed with rt-PCR positive pharyngeal swabs for SARS-CoV-2. In 39 patients, COVID-19 was taken into account as a differential diagnosis (Robert Koch Institute “case in clarification”), and 3 of this subgroup were positive for SARS-CoV-2. Of them, the pharyngeal swab was PCR positive in one case, while in the other 2 cases, only tracheal fluid was PCR positive while the pharyngeal swabs were negative (cases #4 and #5). Overall, a total of 5 SARS-CoV-2 positive patients accounted for 11.9% of all strong and differentially suspected cases and 5.3% of all resuscitation room patients during the study period. Figure 1 outlines the different groups of resuscitation room patients and different outcome numbers regarding SARS-CoV-2 infection of this collective.

Overall, 46/95 patients (48%) required ventilation or securing the airway. Endotracheal intubation was performed in the ED in 29 nontrauma and 7 trauma patients. In 6 of the 29 nontrauma patients, noninvasive ventilation was performed as a salvage attempt. In 10 other nontrauma cases, noninvasive ventilation alone was satisfactory. Of the SARS-CoV-2 positive patients, endotracheal intubation was performed in 2/5 cases, in one of them after an unsuccessful salvage attempt with noninvasive ventilation.

Table 2 outlines the characteristics and diagnostic findings of the SARS-CoV-2 positive patients at ED admission. Some patients were diagnosed with COVID-19 although no typical clinical signs were present at admission or clinical signs were well explained by other pathologies. We present three cases with unexpected diagnostic results in detail.

### 3.1. Cases with Unexpected COVID-19 Diagnoses

#### 3.1.1. Case #3: 70-Year-Old Male Collapse and Cardiac Arrest during Coagulation of Epistaxis—No Clinical Signs of COVID-19, Chest CT Positive, and Pharyngeal Swab PCR Positive

**Figure 2:**
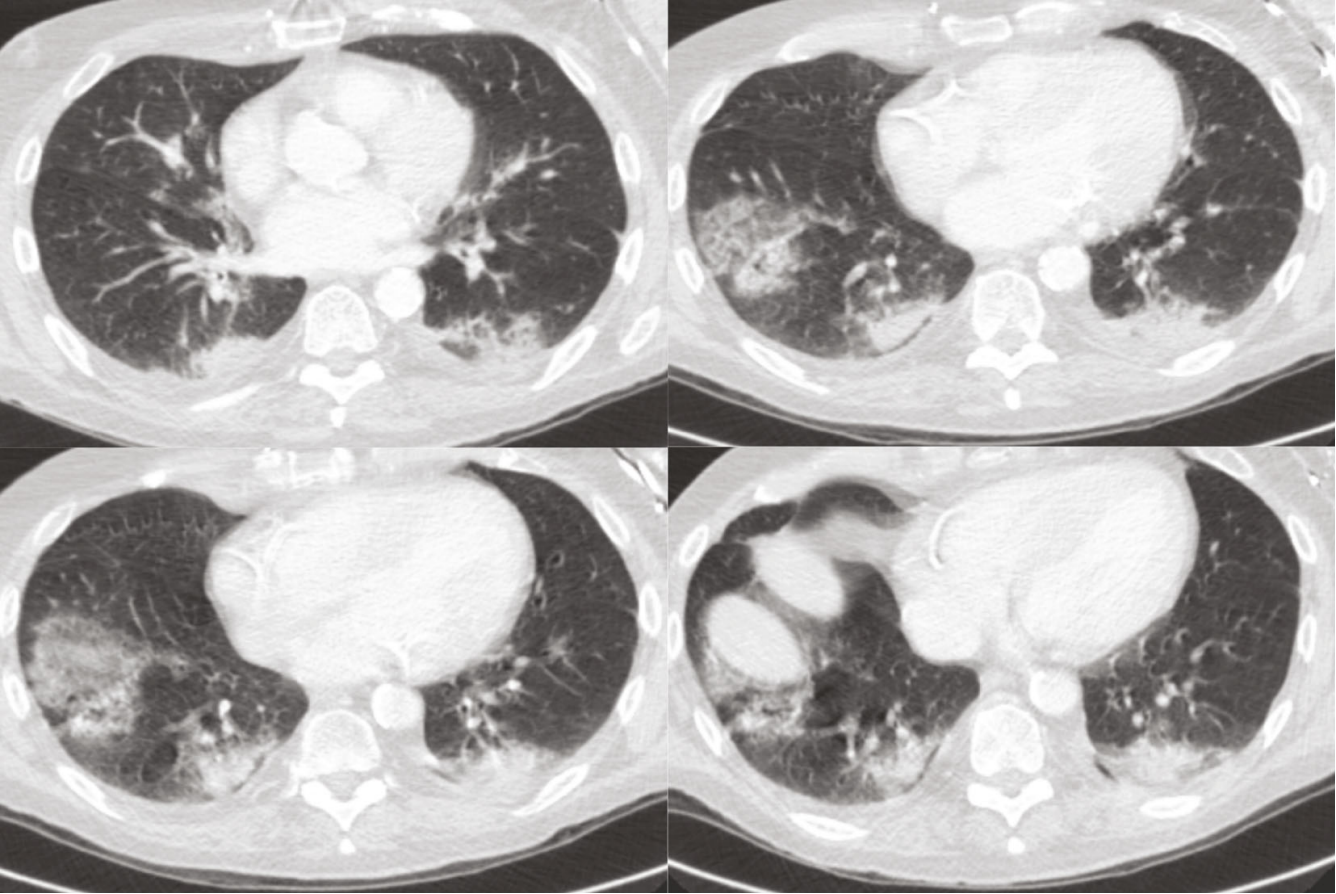
Computed tomography images of case #3.

The patient from a nursing home had an outpatient appointment in the ear, nose, and throat clinic due to recurrent epistaxis. He had no fever or any respiratory complaints. During coagulation in a sitting position, he suddenly collapsed and had a respiratory arrest and no palpable pulse. Cardiopulmonary resuscitation (CPR) with chest compressions and bag-valve-mask was started immediately, and the in-hospital medical emergency team was summoned. When the team arrived 3 minutes later, the patient was already breathing spontaneously and had a palpable carotid pulse. The patient was transferred to the ED resuscitation room and handed over to the ED team. After primary survey, 12-lead-ECG (no signs of myocardial ischemia), focused assessment with sonography of abdomen and thorax (E-FAST) and placement of an arterial line, a post CPR computed tomography protocol was carried out including head CT with angiography as well as contrast-enhanced chest and abdominal CT. The only pathologies that were detected were bipulmonal signs of COVID-19 (CO-RADS 5, Figure 2). Due to multiple previous illnesses and advanced dementia, the patient was transferred in stable condition to our standard care ward for COVID-19 patients with a do-not-resuscitate and do-not-intubate decision.

#### 3.1.2. Case #4: 86-Year-Old Female with an Acute Airway Problem at Admission—Chest CT Negative, Pharyngeal Swab rt-PCR Negative, and Tracheal Secretion PCR Positive

**Figure 3:**
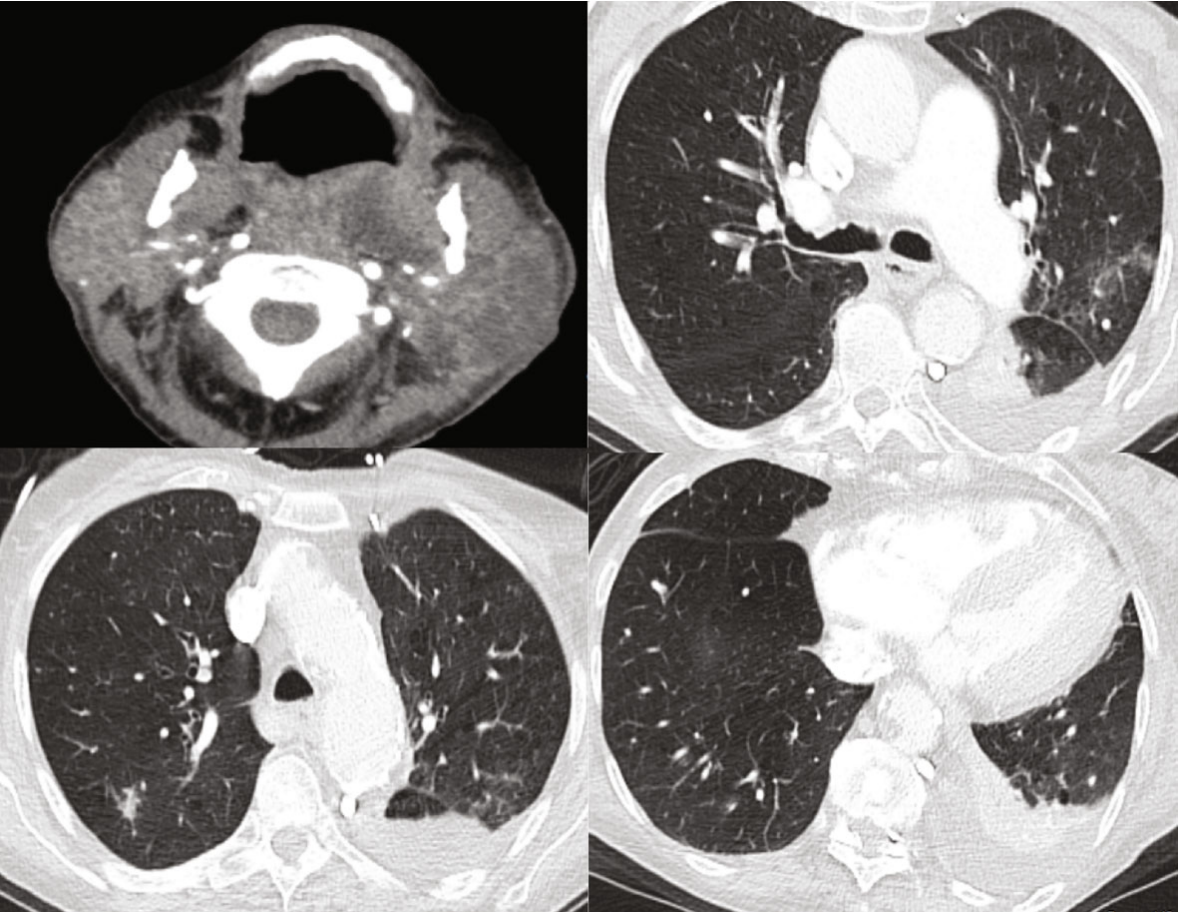
Computed tomography images of case #4.

This 86-year-old female was admitted via EMS from a nursing home with acute respiratory problems (Table 2). During admission, a possible airway obstruction was suspected. She had a history of a pharyngeal abscess, treated with oral amoxicillin since one week. After flexible nasal endoscopy, rapid sequence induction was performed with fentanyl, propofol, and rocuronium, and endotracheal intubation was successful during the first attempt using videolaryngoscopy with McIntosh blade (C-MAC, Karl Storz, Tuttlingen, Germany). After circulatory stabilization with norepinephrine, placement of an arterial line, sampling, and administration of piperacillin/tacobactam, contrast-enhanced head CT, neck CT, and chest CT were performed. The neck CT confirmed the parapharyngeal abscess (Figure 3), and the chest CT showed pulmonary embolism, a pulmonary abscess and no explicit signs of COVID-19 (CO-RADS 2, Figure 3).

At this time point, the signs of infections were well explained by the abscesses, and hypoxia was also well explained due to pulmonary embolism. In the morning of the next day, rt-PCR results of the initial pharyngeal swab were available and showed no SARS-CoV-2 infection. The patient was still isolated due to pending PCR results of tracheal secretion taken after ICU admission. This specimen was SARS-CoV-2 positive although chest CT showed no signs of ground-glass opacities (Figure 3, signs of infections other than COVID-19—CO-RADS 2).

#### 3.1.3. Case #5: 76-Year-Old Male with Respiratory Insufficiency and Cardiogenic Shock due to Myocardial Infarction

The patient was admitted to the resuscitation room via EMS due to hypoxia, dyspnea, shock, and signs of myocardial ischemia. Endotracheal intubation with midazolam, fentanyl, and rocuronium was performed. After circulatory stabilization and contrast-enhanced head and thorax CT (CO-RADS 3), the patient was transferred to catheter laboratory. The pharyngeal swab for SARS-CoV-2 was negative, and tracheal fluid was positive. After extubation, he suffered from acute stroke on day 4 and died due to acute circulatory arrest and unsuccessful cardiopulmonary resuscitation.

## 4. Discussion

To our knowledge, this is the first study presenting a collective of critically ill patients in a German COVID-19 hot-spot region during the pandemic outbreak in 2020. It demonstrates that resuscitation room management during the COVID-19 outbreak was challenging and led to unexpected positive results of SARS-CoV-2 infections, not fulfilling the published suspect case criteria by the Robert Koch Institute. The total number of SARS-CoV-2 positive critically ill patients was considerably lower than initially expected, but still in 12% of all suspected cases, SARS-CoV-2 infection was confirmed, although we used intentional overtriage regarding COVID-19 suspicion. Overall, a high rate of patients requiring mechanical ventilation was observed during this period. Clinical signs like fever and respiratory symptoms alone seemed to be no good sole predictors of SARS-CoV-2 infection, which is shown in our detailed case descriptions and in Table 2. In contrast, a recent report from an U.S. describes fever in 94% of the COVID-19 patients at ED admission [8]. The patient collectives described by other authors in different settings also show a wide range of different symptoms and laboratory findings [1–4, 9, 10]. The heterogeneity of symptoms and initial clinical and laboratory findings is also reported by Goyal et al. in New York City [9]. Elevated D-dimers and lymphopenia are frequently described laboratory findings, but they are no safe screening tool for COVID-19 suspicion which is supported by our cases (Table 2) [3, 4]. Suspicion of COVID-19 cannot be belayed on a few parameters, but all information and clinical findings have to be included. In our case #4, there was a risk of too early deisolation due to findings that supported other reasons for fever and hypoxia and a negative pharyngeal swab rt-PCR. To minimize the risk of false negative results, we recommend testing tracheal fluid with rt-PCR when the combination of routine laboratory results is suggestive of COVID-19 (e.g., lymphopenia, elevated D-dimers, and elevated white blood count). This security strategy is also recommended by Ferrari et al. as a result of their ED patient collective in Italy [11]. In a case series, reported by Wee et al., approximately 40% of confirmed COVID-19 cases of the ED showed no typical clinical signs of COVID-19 [12]. Our strategy of expanded suspicion criteria is also supported by their results. During the outbreak of COVID-19 in Italy, the incidence of out of hospital cardiac arrest increased considerably in the Lombardi region [13]. Therefore, it could be assumed that cardiac arrest due to COVID-19 is more frequently than confirmed. Personal protective equipment should be used in every cardiopulmonary resuscitation like in our presented case with an unremarkable initial medical history regarding COVID-19.

Our study has all well-known limitations of retrospective analyses. However, inclusion of patients as “resuscitation room cases” was prospectively and independently due to an already established inclusion process in the context of the local quality management. Nevertheless, we cannot exclude missing cases due to forgotten questionnaires. Our number of SARS-CoV-2 positive patients could be also false too low, because not all 95 patients were tested for SARS-CoV-2. Although this seems to be a relevant limitation, other retrospective studies and case series in Chinese and U.S. hospitals show the same limitations regarding the fraction of SARS-CoV-2 positive patients [1–3, 9].

## 5. Conclusion

In emergency medicine, there is often a knowledge gap regarding the medical history and clinical signs that led to deterioration. Discrimination for COVID-19 based only on clinical signs was found to be difficult in critically ill patients, and also, false negative results for pharyngeal swabs were found. In every resuscitation room patient, we recommend full personal protection and a pharyngeal swab as well as a SARS-CoV-2 rt-PCR of tracheal fluid in intubated patients to prevent false negative results that lead to false deisolation in the ICU. Recent recommendations should be expanded accordingly [14].

## Figures and Tables

**Table 1:** Characteristics of all resuscitation room patients at emergency room admission.

Parameter/characteristic	Number/value
Age (years, mean ± SD), *n* = 94	66 ± 18
Female (%), *n* = 94	41 (43%)
Glasgow coma scale (3-15), *n* = 94	Median: 14; IQR: 6
Respiratory rate/min (mean ± SD), *n* = 64	22 ± 2.3
Oxygen saturation % (mean ± SD), *n* = 72	93 ± 7.3
Body temperature °C (mean ± SD), *n* = 71	37 ± 1.4
Heart rate/min (mean ± SD), *n* = 74	95 ± 31
Systolic noninvasive blood pressure mmHg (mean ± SD), *n* = 69	145 ± 38
Leukocyte count, *n* = 94 (white blood count)	13456 ± 11110
pO_2_ (capillary and arterial, mean ± SD) mmHg, *n* = 69	122 ± 96
pCO_2_ (venous, capillary and arterial, mean ± SD), *n* = 90	43 ± 22
Lactate mmol/l (venous and arterial, mean ± SD), *n* = 89	2.9 ± 2.9

IQR: interquartile range; SD: standard deviation.

**Table 2:** Clinical characteristics of SARS-CoV-2-infected resuscitation room patients at admission.

Parameter/clinical feature	Patient #1	Patient #2	Patient #3	Patient #4	Patient #5
Grade of COVID-19 suspicion	High	High	Low	Low	Low
Age (years)	55	60	70	86	76
Sex	Female	Male	Male	Female	Male
Glasgow coma scale	15	15	10	13	7
SpO_2_ %	96	100	92	96	70
Respiratory rate/min	20	19	20	28	38
Temperature °C	38.5	38.7	37.2	36.4	36.7
Heart rate/min	87	85	80	57	70
Noninvasive blood pressure Systolic	150	166	198	170	88
pO_2_ (arterial or capillary) mmHg	80.5 (arterial)	N/A	119 (arterial)	73.5 (arterial)	44.3 (arterial)
pCO_2_ (arterial/capillary/venous) mmHg	34.1 (arterial)	48.3 (venous)	25.4 (arterial)	38.6 (arterial)	61.8 (arterial)
Lactate (mmol/l)	0.8	1.5	3.6	1.4	1.8
Lactate dehydrogenase U/l	225	535	521	391	1164∗
Elevated D-dimers	No	No	Yes	Yes	Yes
D-dimers (mg/l)	0.59	0.9	35.2	4.88	2.09
White blood count	4120	9490	14300	17600	17940
Lymphopenia (count)	No	No	Yes	Yes	No
Lymphocyte count	1110	1100	580	890	3330
Lymphopenia (relative %)	No	Yes	Yes	Yes	No
Lymphocyte count %	64.4	11.6	4	5	18.6
CRP (mg/dl)	2.7	29.6	22.7	30.8	18.2
PCT (ng/ml)	0.02	0.17	1.03	0.94	0.11
Chest CT (CO-RADS)	4	4	5	2	3

SpO_2_: pulse oxymetry; O_2_: oxygen; CO_2_: carbondioxide; ∗1st sample was hemolytic, value of second sample; CRP: C-reactive protein; PCT: procalcitonin; CT: computed tomography.

## Data Availability

The original data is part of the electronic health records of the Kliniken Maria Hilf Hospital, Mönchengladbach, Germany. Therefore, data is not accessible for the public. Any questions about the analyzed data can be addressed to the corresponding author.
